# NeuroNasal: Advanced AI-Driven Self-Supervised Learning Approach for Enhanced Sinonasal Pathology Detection

**DOI:** 10.3390/s25082369

**Published:** 2025-04-08

**Authors:** Nesrine Atitallah, Safa Ben Atitallah, Maha Driss, Khalid M. O. Nahar

**Affiliations:** 1 Faculty of Computer Studies, Arab Open University, Riyadh 11681, Saudi Arabia; 2 Robotics and Internet-of-Things Laboratory, Prince Sultan University, Riyadh 11586, Saudi Arabia; mdriss@psu.edu.sa; 3 RIADI Laboratory, University of Manouba, Manouba 2010, Tunisia; 4 Computer Science Department, Faculty of Information Technology and Computer Sciences, Yarmouk University, Irbid 21163, Jordan; khalids@yu.edu.jo

**Keywords:** AI, self-supervised learning, random forest, sinonasal, pathology detection

## Abstract

Sinus diseases are inflammations or infections of the sinuses that significantly impact patient quality of life. They cause nasal congestion, facial pain, headaches, thick nasal discharge, and a reduced sense of smell. However, accurately diagnosing these diseases is challenging due to multiple factors, including inadequate patient adherence to pre-diagnostic protocols. By leveraging the latest developments in Artificial Intelligence (AI), there exists a substantial opportunity to improve the precision and effectiveness of classification of these diseases. In this study, we present a novel AI-based approach for sinonasal pathology detection, using Self-Supervised Learning (SSL) techniques and Random Forest (RF) algorithms. We have collected a new diagnostic imaging dataset, which is a major contribution to this study. The dataset contains 137 CT and MRI images meticulously labeled by expert radiologists, with two classes: healthy and unhealthy (sinus disease). This dataset is a useful asset for developing and evaluating AI-based classification techniques. In addition, our proposed approach employs the Deep InfoMax (DIM) model to extract meaningful global and local features from the imaging data with a self-supervised method. These features are then used as input for an RF classifier, which effectively distinguishes between healthy and sinonasal pathological cases. The combination of both DIM and RF provides efficient feature learning and powerful classification of sinus cases. Our preliminary results demonstrate the efficiency of the proposed approach, which achieves a mean classification accuracy of 92.62%. These findings highlight the potential of our AI-based approach in improving sinonasal pathology diagnosis.

## 1. Introduction

Sinus diseases affect millions of people around the world, with chronic rhinosinusitis (CRS) affecting approximately 12% of adults around the world [1]. These conditions encompass a diverse spectrum of disorders that affect the nasal passages and sinus cavities, resulting in significant morbidity and quality-of-life impairment. These disorders include sinusitis, rhinitis, nasal polyps, sinus tumors, deviated nasal septum, various sinus infections, sinus headaches, barosinusitis, Samter’s Triad, mucoceles, and sinus cysts. Effective management of these conditions requires a multidisciplinary approach that integrates innovative research, clinical guidelines, and advanced diagnostic tools [2]. The widespread prevalence of sinus diseases, combined with the substantial economic burden of over USD 8 billion annually in direct costs, underscores the urgent need for more efficient diagnostic and treatment strategies [3].

Current diagnostic techniques for sinus disease are based mainly on conventional methods such as imaging and clinical assessment. However, these methods have raised significant limitations, including frequent subjectivity, interpretation variability, and challenges with subtle pattern recognition.

To overcome these constraints, AI has been used, in particular Machine Learning (ML) and Deep Learning (DL) [4,5,6]. In fact, AI applications in healthcare have been transformative, as discussed in [4], where AI’s impact spans various domains, including medical imaging and diagnostics, virtual patient care, drug discovery, and rehabilitation. Beyond general advances in AI-driven healthcare, Federated Learning (FL) has emerged as a promising approach to preserve patient privacy while enabling collaborative model training across distributed healthcare institutions. Hossain et al. [5] demonstrated the effectiveness of FL in cancer classification using histopathological images of lung and colon cancers. Furthermore, Atitallah et al. [6] addressed the scarcity of labeled data by using Few-Shot Learning (FSL) and ensemble learning to enhance Alzheimer’s disease detection. Furthermore, Abdel-Jaber et al. [7] applied ML to stroke detection, evaluating six classification models with various feature selection methods. Decision tree (DT) emerges as the most effective algorithm, achieving the highest accuracy, precision, recall, and F1-score. Amanian et al. [8] reviewed AI applications in rhinology, analyzing 59 studies in areas such as diagnostics, rhinosinusitis classification, and surgical navigation. They highlight AI’s growing role since 2016 and stress the need for validation and clinical integration. Both studies show the impact of AI on medical diagnostics, with work [7] advancing stroke prediction and work [8] highlighting the potential of AI in rhinology and surgery.

Building on these advances, it is clear that AI is emerging as a transformative force across multiple areas of healthcare. Using advanced algorithms to analyze vast amounts of patient data, AI is improving diagnostic accuracy, personalizing treatment plans, and streamlining clinical workflows [9,10]. This integration of AI in healthcare care, particularly in the management of sinus diseases, promises to revolutionize patient care by improving early detection, treatment efficacy, and access to specialized medical expertise [11].

ML techniques have become increasingly popular in healthcare, with RF emerging as a powerful tool for classification and regression tasks [12,13]. RF is an ensemble learning method that constructs multiple decision trees during training and outputs the class, that is, the mode of the classes (classification) or mean prediction (regression) of the individual trees. Its strengths lie in its ability to handle high-dimensional data, resistance to overfitting, and the ability to classify feature importance.

Moving beyond traditional supervised learning, SSL has been introduced as an advanced ML method that has gained significant attention in recent years [14]. SSL employs artificially supervised tasks to learn meaningful representations from unlabeled input. Large volumes of unlabeled data are used by SSL in medical imaging to pre-train models, which are then refined on smaller labeled datasets [15]. This approach is mainly useful in healthcare, where labeled data are scarce and expensive to acquire. By capturing hierarchical and spatially-aware visual features, SSL facilitates robust feature extraction and enhances generalization across diverse diagnostic tasks without relying on extensive manual labeling.

In this paper, we propose a novel approach named NeuroNasal for accurate and efficient detection of sinonasal pathology. NeuroNasal integrates SSL with RF to address the challenges of limited labeled data and complex feature extraction in medical imaging. Using SSL, the model effectively learns rich and meaningful representations from large amounts of unlabeled data, enhancing its ability to capture intricate patterns and structures within CT and MRI images. The RF classifier then utilizes these extracted features to perform a precise and robust detection, distinguishing between healthy and diseased cases.

The main contributions of this paper are as follows:

- Collection of a diagnostic imaging dataset comprising 137 CT and MRI images expertly labeled into healthy and unhealthy (sinus disease) categories developed for sinonasal pathology detection.
- Development of a novel hybrid learning framework that specifically addresses sinonasal pathology detection challenges by combining SSL-based feature extraction with RF classification, allowing effective learning from limited labeled medical data.
- Addressing the challenge of limited labeled data in this domain by implementing a hybrid learning approach that combines SSL for efficient feature extraction from unlabeled sinonasal images with RF classification.

The remainder of this paper is structured as follows. Section 2 presents a comprehensive review of the literature on the classification of sinus diseases. Section 3 explores the underlying theoretical concepts. Section 4 investigates the proposed methodology. Section 5 elaborates on the dataset used in our study. Section 6 explains the experimental results and their implications. Section 7 provides a detailed discussion of the findings, including limitations and implications for future work. Finally, Section 8 presents the concluding remarks and outlines future directions for research in this domain.

## 2. Related Works

In this section, we provide a comprehensive review of related work, highlighting key challenges and limitations. Many studies highlight AI’s potential in diagnosing various diseases through clinical data and image analysis. These studies emphasize the AI’s ability to detect micro-features beyond human capacity.

Ding et al. [16] demonstrated the successful application of advanced ML algorithms like ResNet, InceptionV3, and Unet in Otitis Media (OM) diagnosis. They highlighted the potential for an early and accurate diagnosis, as these are powerful tools in image recognition and segmentation.

Furthermore, Patil et al. [17] represented significant advancements in the application of AI in dental implantology. The planning of maxillary sinus floor augmentation procedures was thoroughly explored. The authors anticipated significant advances in dental diagnostics in the next decade, but emphasized the need to overcome current limitations to successfully integrate them into routine dental practice.

AI has been used in diagnosing various sinus diseases through clinical data and image analysis. Rhinosinusitis (RS), a common inflammatory condition affecting the sinonasal mucosa and paranasal sinuses, was deeply explored by Huang et al. [18]. In addition, various pathogenic microorganisms, including viruses, bacteria, and fungi, and their mechanisms that cause RS were involved and discussed. Massey et al. [19] evaluated a novel automated CT analysis system using convolutional neural networks for the diagnosis of CRS. The research, involving 88 adult patients, demonstrated successful automated segmentation of CT scans in various populations and imaging systems. Although the system successfully provided objective quantification of sinus opacification and demonstrated potential for standardized assessment, the authors note that further validation in prospective, multi-institutional settings would be beneficial for broader implementation.

Huang et al. [20] developed automatic classification surgical plans for augmentation of the maxillary sinus floor in dental implant procedures. The model, named SinusC-Net, employed a two-stage approach: detection, which automatically identifies five landmarks on Cone Beam Computed Tomography (CBCT) images, and then classification, which categorizes images into five surgical approaches using a 3D distance-guided network. The results showed high accuracy in terms of landmark detection: Mean error of 0.87 mm, with a 95.47% success rate within 2 mm. Furthermore, the classification performance was high with 97% accuracy, 92% sensitivity, 98% specificity, and 95% Area Under the Curve (AUC).

He et al. [21] represented a significant advance in the treatment of CRS, particularly for patients requiring surgical intervention. The authors developed a nomogram based on DL. This approach is used to preoperatively predict recurrence in patients with CRS that require surgical treatment. They achieved promising results, but they also candidly acknowledge several limitations, which is commendable in scientific research. These limitations revolve mainly around the heterogeneity of the data, sample size, and the interpretability of the DL models.

Bu et al. [22] developed a computer-aided diagnostic system to assist clinicians, especially general practitioners in primary hospitals, in the differential diagnoses of sore throat. The validation process appears thorough, with initial testing, third-party verification, and real-world application. The high accuracy rates and clinician acceptance are particularly noteworthy. Although this study addresses a significant challenge in primary care, it highlights misdiagnosis due to limited specialist knowledge and experience.

Liu et al. [23] developed a specific patient method, which is a learning-based method to reconstruct the anatomy of the sinus surface directly and only from endoscopic videos. Their approach demonstrated its ability to work directly from endoscopic videos without requiring additional imaging modalities, which is a major advantage. As future directions, this study recommends exploring more complex network architectures (e.g., self-supervised recurrent neural networks).

Doğan and Bor [24] proposed a novel automation and decision support system for the classification of Gastroesophageal Reflux Disease (GERD) and potentially the classification of sinus disease. They implemented ML algorithms for decision support. The automatic phenotyping feature helps standardize diagnosis and treatment approaches, which can be especially beneficial for less experienced physicians. This study demonstrates the potential of AI and ML in improving both clinical practice and research in gastroenterology. Table 1 summarizes the key characteristics of some related works, highlighting their approaches, datasets, and performance metrics in sinonasal pathology detection.

From Table 1, recent advances in AI and ML have shown promising results in various areas of medical imaging and diagnosis. However, the application of these technologies to sinus disease detection is still in its early stages and presents several challenges and opportunities for improvement.

Despite these advancements, several key gaps and limitations in current research have been identified:

- Data scarcity: There is a lack of high-quality, labeled medical datasets, especially for sinus diseases. This limitation hinders the development and validation of AI models.
- Integration into clinical practice: Although many studies show promising results, there is still a gap in the successful integration of these AI tools into routine clinical practice.
- Multidisciplinary approach: More research is needed to explore the connections between different conditions (e.g., RS and asthma) and develop more holistic AI-based diagnostic and treatment approaches.
- Addressing misdiagnosis: There is still a need to improve AI systems to reduce misdiagnosis rates, especially in primary care settings where specialist knowledge may be limited.
- Advanced AI approaches: There is a need to explore and implement more sophisticated AI techniques, particularly SSL. This approach could potentially address some of the data scarcity issues by allowing models to learn from unlabeled data, which is often more abundant in medical settings.

Addressing these gaps could significantly advance the field of AI in medical diagnostics, particularly for sinus and related diseases, leading to more accurate, efficient, and accessible healthcare solutions. The field remains open for further exploration and refinement as research progresses and technologies mature.

## 3. Theoretical Backgrounds

This section explores the underlying concepts and theories driving the integration of AI into healthcare. It also delves into SSL, an ML approach that leverages large amounts of unlabeled data to learn meaningful representations.

### 3.1. AI in Healthcare

AI has revolutionized healthcare significantly by improving the quality and efficiency of patient care. One of the key contributions of AI in the healthcare sector is its ability to enable precise diagnosis and reliable pathology detection [7]. Advanced AI models can analyze medical images, laboratory data, and electronic health records to detect diseases such as cancer, neurological disorders, and cardiovascular diseases, with greater speed and precision than traditional methods [10]. This leads to earlier detection and allows rapid interventions to improve patient outcomes [27].

A significant recent development is the rise of Explainable AI (XAI). XAI focuses on improving transparency in AI-driven decision-making processes, addressing critical issues around trust and accountability. By making AI decisions more interpretable and understandable for medical professionals, XAI helps foster confidence and promotes a wider adoption of AI technologies in clinical settings [28].

Kitsios et al. present a comprehensive review of 132 academic publications that underscores the progress of AI in healthcare care, emphasizing its role in improving diagnostic procedures, early disease detection, and optimization of the healthcare system [29]. Despite these advances, integrating AI into healthcare is not without challenges. The issues surrounding data privacy, algorithmic biases, and the need for robust regulatory frameworks are key hurdles that need to be addressed [30].

Together, these studies illustrate significant progress and challenges in the integration of AI into healthcare systems. They emphasize the potential of AI to revolutionize patient care while highlighting the importance of addressing ethical, regulatory, and privacy concerns. Among emerging AI techniques, SSL has been presented as a powerful approach, offering significant potential to address some of these challenges by using unlabeled data for effective model training.

Recent studies highlight the transformative role of AI in healthcare, showing its diverse applications in diagnosis, personalized treatment, and patient-care coordination [31]. The advent of Explainable AI (XAI) is particularly significant, as it aims to improve transparency in AI-driven decision making processes, thus building confidence among medical professionals [28]. Talati et al. [32] expand on the potential of AI in predictive analytics and early disease identification, highlighting the need for comprehensive regulatory structures to optimize its impact. A comprehensive analysis of 132 academic publications reveals considerable progress in AI applications in healthcare, with a focus on early detection, diagnostic procedures, and system optimization [29]. This review underscores both the advantages and challenges associated with the deployment of AI technologies in clinical environments. However, the integration of AI in healthcare faces several hurdles, including concerns about data privacy, algorithmic bias, and the need for robust regulatory frameworks [30]. Collectively, these investigations demonstrate significant progress and challenges in incorporating AI into healthcare systems, emphasizing its potential to revolutionize patient care while underscoring the importance of addressing ethical, regulatory, and privacy concerns.

### 3.2. Self-Supervised Learning

**Figure 1:**
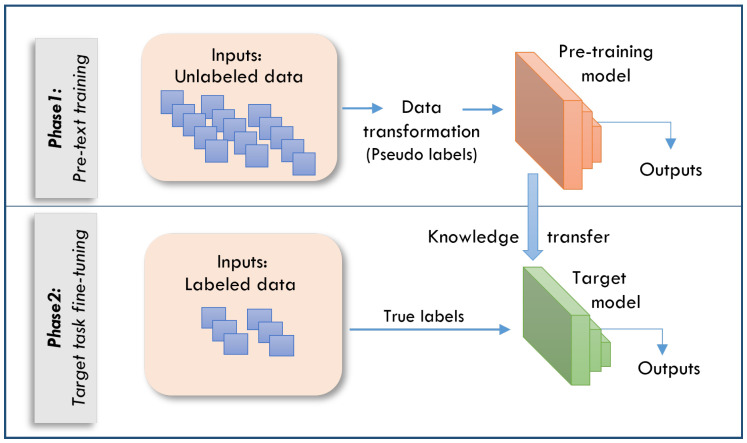
The two phases of the SSL framework.

In healthcare, SSL has gained prominence as an approach to drive valuable data representations without the need for extensive labeled datasets [14,33]. SSL diverges from conventional supervised learning methodologies by generating its own training signals from the data rather than relying on human-annotated labels. This process involves the creation of pretext tasks that enable the model to learn meaningful representations. These learned representations can be subsequently applied to various downstream tasks, including classification, detection, and segmentation. The SSL framework typically operates in two distinct phases, as shown in Figure 1:

- Pretext task: The model learns representations from unlabeled data.
- Transfer to downstream tasks: The learned representations are used for specific medical applications.

This approach offers significant potential in healthcare, where labeled data can be scarce or expensive to obtain.

SSL techniques can be categorized into three main approaches [34]:

- Contrastive Learning: This method learns by drawing similar examples (positive pairs) together while separating dissimilar examples (negative pairs) in the representation space.
- Predictive Learning: This approach involves learning by predicting missing or future components of the data, as seen in autoregressive models.
- Generative Learning: This technique learns by creating new data that mimic the original dataset, exemplified by Variational AutoEncoders (VAEs) and Generative Adversarial Networks (GANs).

Among these, contrastive learning has shown exceptional potential in developing high-performance representations in various fields, including vision, language, and medical applications [35]. Contrastive learning, a subset of SSL, focuses on learning data representations by contrasting positive and negative sample pairs. Its primary goal is to minimize the distance between positive pair representations in the latent space while maximizing the distance between negative pairs. In this context, positive pairs represent different views, transformations, or augmentations of the same data sample, whereas negative pairs consist of unrelated samples.

DIM is an innovative SSL framework that aims to create robust data representations by optimizing the mutual information between the global and local data features [36]. In contrast to conventional contrastive methods that compare augmented versions of the same data, DIM’s approach involves contrasting the global representation of the entire input against local representations derived from specific data subsets. The primary objective of DIM is to maximize the mutual information between two key components:

- A global feature vector that encapsulates the entire input.
- Local feature vectors extracted from specific regions or segments of the input.

This approach is founded on the principle that an effective representation should simultaneously capture both the overarching context and the granular details of the data. In doing so, DIM aims to create more comprehensive and nuanced data representations that can be leveraged for various downstream tasks.

## 4. Proposed Approach: NeuroNasal for Enhanced Sinonasal Pathology Detection

**Figure 2:**
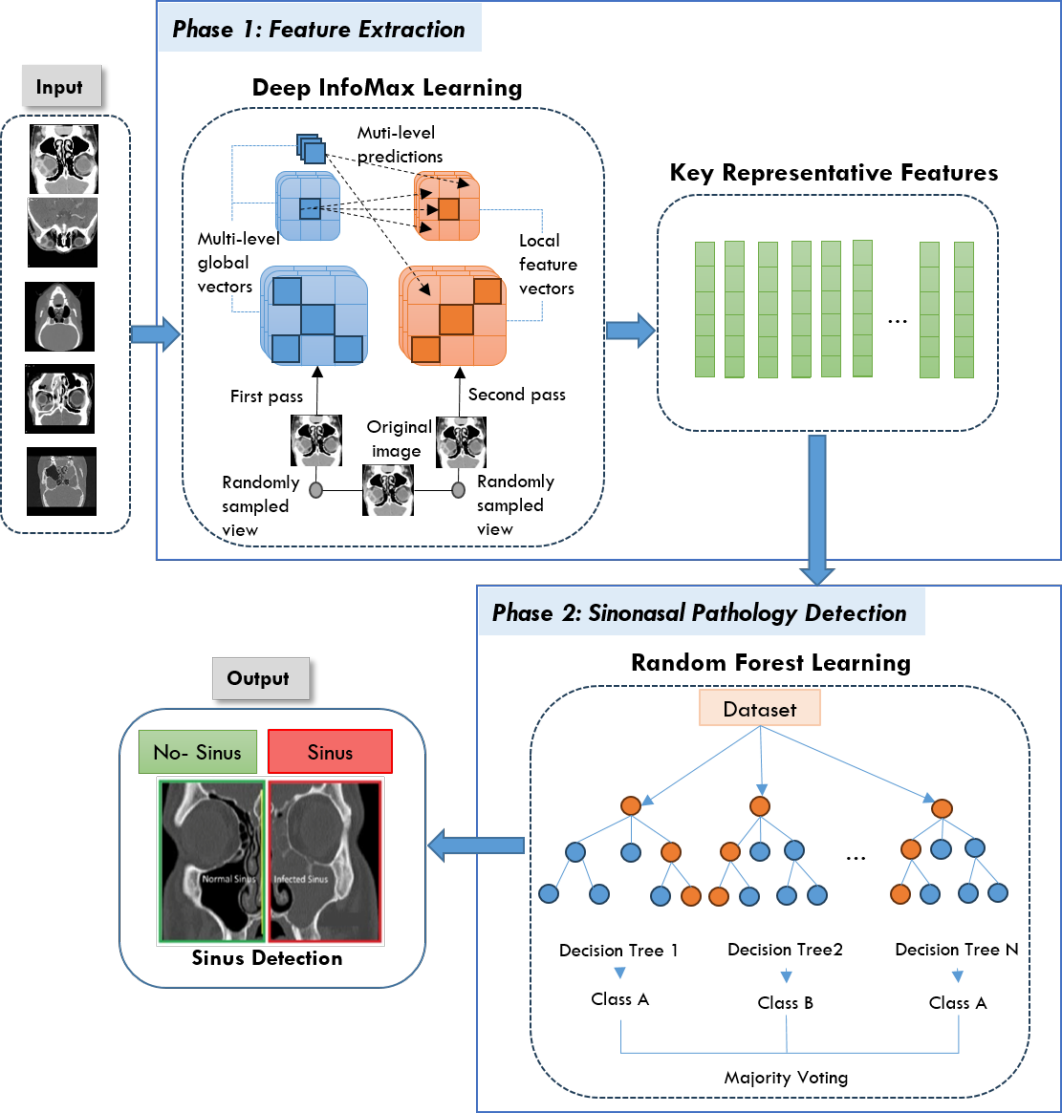
The architecture of the proposed NeuroNasal approach.

Our goal in this study was to create NeuroNasal, a powerful approach to detect sinus diseases that combines the advantages of SSL with conventional ML classifiers. The proposed approach addresses the challenges of existing classification systems by improving feature extraction. It integrates DIM for self-supervised representation learning and an RF classifier for effective classification without requiring extensive labeled datasets. NeuroNasal consists of two main phases: SSL for feature extraction and classification using RF. The architecture of the proposed approach is shown in Figure 2. The process begins with a raw dataset consisting of medical CT scans of the sinus region, including images of the normal and infected sinus. Raw CT images serve as input, and DIM learning extracts robust global and local features through a two-pass process. These features are condensed into key representative data points for classification. The RF classifier, using ensemble decision trees, predicts healthy or unhealthy, with results visually highlighted. This approach ensures accurate and efficient detection, supporting clinical diagnostics. More details about each phase of the proposed approach are discussed in the following subsections.

### 4.1. Phase 1: Feature Extraction

In this phase, DIM was selected as the SSL method due to its ability to learn rich representations by maximizing mutual information between local and global features within an image. This property is particularly advantageous for medical imaging tasks, where critical diagnostic information is spatially localized and subtle in nature. Unlike contrastive methods such as SimCLR, MoCo, and BYOL, which require large batch sizes, complex augmentation pipelines, or momentum encoders, DIM offers a relatively simple but effective framework that performs well in small-scale datasets [37]. Moreover, DIM does not rely on negative samples or large computational resources, making it a practical choice for the medical environment. Its architecture is well suited to capture the contextual relationships within medical images, which is essential to distinguish between healthy and pathological regions in sinonasal imaging.

Firstly, we extract meaningful feature representations from the input data using the DIM model, an SSL contrastive learning method. By maximizing mutual information I(y,M) between global representations *y* and local patches *M* within each image, the model learns both global and local characteristics, allowing high-quality feature extraction without the need for labeled data — a key advantage for unsupervised learning tasks.

Let $x=\{x_{1},x_{2},\ldots ,x_{n}\}$ represent the input feature set, where each $x_{i}$ is a feature vector extracted from the input data. The objective of the DIM model is to maximize I(y,M), ensuring that the most informative aspects of the input data are retained in the global representation, as defined in Equation (1).

(1) $$\max I\left(y,M\right)=\max E_{p(y,M)}\left[\log\frac{p(y,M)}{p(y)p(M)}\right]$$

where p(y,M) is the joint probability distribution of the global representation *y* and the local patch *M*, and p(y) and p(M) are the marginal distributions of *y* and *M*, respectively.

To facilitate optimization, mutual information is approximated through a lower bound objective using a discriminator, which distinguishes between the joint distribution (y,M) and the product of marginals p(y)p(M).

Once the DIM model extracts both global and local feature representations, these learned representations are used as input for the RF classifier.

### 4.2. Phase 2: Sinonasal Pathology Detection

In the classification phase, the learned representations from the DIM model are passed to an RF classifier. The RF model, known for its robustness and ability to handle high-dimensional data, is used to perform the final classification. The classifier is trained on the encoded features as shown in Figure 2.

We selected RF due to its proven effectiveness in modeling nonlinear relationships and its strong performance on small to medium-sized datasets. It is less sensitive to overfitting and requires minimal hyperparameter tuning. Furthermore, RF is particularly well suited to the self-supervised feature representations generated by DIM, as ensemble models often perform well when provided with rich, high-level encodings [38].

The RF classifier is used as an ensemble method to combine predictions from multiple decision trees. Given a set of features $x=\{x_{1},x_{2},\ldots ,x_{n}\}$, where each $x_{i}$ represents a feature of the input data, the RF constructs *M* decision trees to make predictions.

Each tree $m_{j}$ predicts a class label $p_{j}\left(x\right)$ from the set of possible labels Y. The final classification is determined using majority voting across the ensemble of trees. The final output f(x) is given by:

(2) $$f\left(x\right)=\arg\max_{y\in Y}\sum_{j=1}^{M}I\left(y=p_{j}\left(x\right)\right)$$

where f(x) is the final predicted class label, *y* represents a possible class label from the set Y, *M* is the total number of decision trees in the RF, $p_{j}\left(x\right)$ is the predicted class from the *j*-th decision tree, and $I(y=p_{j}\left(x\right))$ is an indicator function that returns 1 if the prediction of the *j*-th tree is *y*, and 0 otherwise.

In the case of probability-based voting, each tree $m_{j}$ produces a probability distribution on the class labels. The final probability P(y|x) for class *y* is calculated using Equation (3):

(3) $$P\left(y|x\right)=\frac{1}{M}\sum_{j=1}^{M}P_{j}\left(y|x\right)$$

where $P_{j}\left(y|x\right)$ is the probability predicted by the *j*-th tree that the input belongs to class *y*.

After that, the class with the highest average probability is chosen to be the final class label, following Equation (4).

(4) $$f\left(x\right)=\arg\max_{y\in Y}P\left(y|x\right)$$

## 5. Dataset

**Figure 3:**
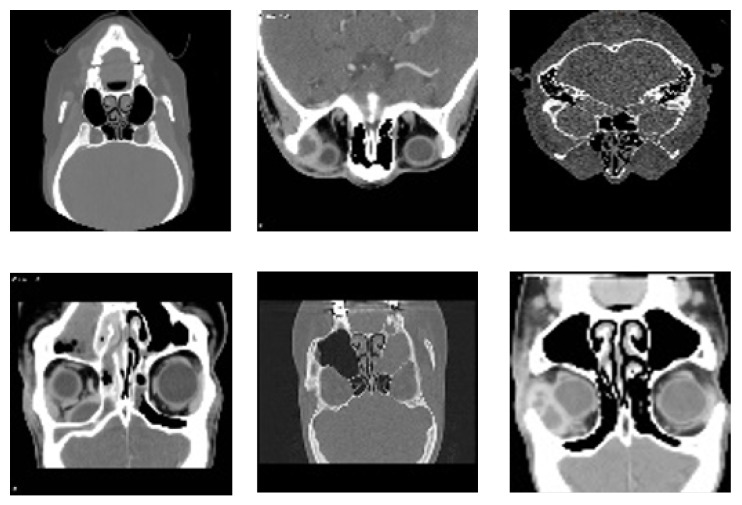
Representative samples from the dataset, showcasing diverse imaging types and anatomical views of sinonasal regions used for training and validation of the proposed detection model.

The Radiopaedia website [39] provides a diverse collection of cases featuring extensive imaging studies, including CT scans and MRIs from multiple individuals, each representing a unique subject. All images have been carefully annotated by radiologists, making this repository a valuable resource for both research and educational purposes. These datasets offer critical insights into diagnosis and classification. However, as highlighted in [40], publicly available datasets on sinus diseases remain scarce. To address this gap, we undertook a rigorous effort to filter and refine a dataset specifically focused on sinus pathologies. Our approach involved systematically reviewing the extensive Radiopaedia repository [39], which contains images related to various medical conditions. We carefully identified, categorized, and extracted only the most relevant and high-quality sinus-related images. This meticulous curation ensured that our dataset is both comprehensive and specifically tailored to sinus pathologies, enhancing its utility for research in AI-assisted diagnosis. Furthermore, to facilitate further advancements in the field, we have made this dataset publicly available for all research purposes in [41]. Figure 3 illustrates samples from the dataset.

The data collected from the website [39] are distributed across two classes: healthy, consisting of 37 instances, and unhealthy, consisting of 100 instances, for a total of 137 samples. Imaging data were collected from 137 participants, with scans obtained from multiple clinics to ensure diversity in the analysis.

The diversity of conditions and the inclusion of rare complications make this dataset particularly valuable for developing diagnostic tools for sinus-related conditions.

## 6. Experiments

This section outlines the results of evaluating the proposed NeuroNasal for sinonasal pathology classification. We start by presenting the experimental setup and then we define the evaluation metrics. After that, we provide and discuss the experimental result. In addition, we have compared the proposed approach with various conventional ML and transfer learning models. Finally, the interpretation of the experimental results is provided in the discussion.

### 6.1. Experimental Setup

For experimental tests, we have used a system with the following hardware specifications: Intel(R) Core(TM) i7-8565U CPU @ 1.80 GHz, 16 GB of RAM, and an NVIDIA GeForce MX series GPU, operating on Windows 11. The implementation employed several key ML libraries: the PyTorch (version 1.13.1) [42] for model training and evaluation, the scikit-learn (version 1.2.2) for data pre-processing and performance metrics [43], and Seaborn/Matplotlib (version 3.7.1) [44] for visualizing results. The dataset was divided into 90% for training and 10% for testing.

To ensure input consistency and enhance model generalization, several preprocessing steps were applied to the dataset before training. All images were visually inspected to verify diagnostic quality and ensure baseline clarity. To address the class imbalance (100 unhealthy vs. 37 healthy cases), data augmentation techniques, including random horizontal flipping, small-angle rotations, and zoom operations, were selectively applied to the minority class (healthy) during training. Additionally, all images were resized to a uniform resolution of 224 × 224 pixels and normalized to standardize pixel intensity distributions.

Our approach follows a two-stage process including self-supervised feature extraction and supervised classification with a k-fold cross-validation to ensure robust evaluation. In the self-supervised feature extraction stage, the DIM model is trained using only the training set (90%) without any labeled data. Once the features are extracted, the model transitions to the supervised classification stage, where the encoder is frozen, and the extracted features from the training images are used to train the RF classifier. The test set (10%) remains completely unseen throughout both the feature extraction and classifier training stages, ensuring a strict separation between training and testing to prevent information leakage.

During evaluation, the trained DIM model is used to extract features from the test set, which are then classified using the trained RF classifier. The whole hyper-parameters used in configuring the used models are presented in Table 2.

### 6.2. Evaluation Metrics

The effectiveness of the proposed approach was thoroughly evaluated using a set of crucial performance metrics. The selected metrics include the following.

- Accuracy: This measure indicates the general correctness of the model in all categories, calculated by dividing the number of correct predictions by the total number of predictions made.
  (5) $$Accuracy=\frac{TP+TN}{TP+TN+FP+FN}.$$
- Precision: This metric shows the proportion of correct positive predictions out of all positive predictions made.
  (6) $$Precision=\frac{TP}{TP+FN}.$$
- Recall (Sensitivity): This measure represents the proportion of actual positive cases that were correctly identified.
  (7) $$Recall=\frac{TP}{TP+TN}.$$
- F1-Score: This score balances precision and recall by calculating their weighted average.
  (8) $$F1=2\times \frac{Precision*Recall}{Precision+Recall}.$$
- AUC-ROC (Area Under the Receiver Operating Characteristic Curve): This statistic gives an in-depth overview of the performance of the model at various classification levels. The ROC curve graphically contrasts true and false positive rates, whereas the AUC measures total performance in a single value.

### 6.3. Experimental Results

The suggested NeuroNasal achieved an excellent mean classification accuracy of 92.62%. This shows its high accuracy in discriminating between healthy and sick sinonasal states. Table 3 presents the performance metrics of the proposed approach evaluated using a five-fold cross-validation. It reports accuracy, precision, recall, and F1-score for each fold, along with their mean and standard deviation (Mean ± Std). The reported precision, recall, and F1-score are weighted on average, which means that the contribution of each class to the overall metric is proportional to its frequency in the dataset. This averaging method was selected to account for the inherent class imbalance, providing a more realistic representation of the performance of the model in the majority and minority classes.

We can see from the table that the accuracy values range from 89.5% (Fold 2) to 95.1% (Fold 3), with an overall mean of 92.62% ± 2.24. The precision ranges from 88.9% to 94.3%, with an overall mean of 91.88% ± 2.15. On the other hand, the recall varies between 89.2% and 95.0%, averaging 92.36% ± 2.27. The F1-score is consistent, ranging between 89.0% and 94.6%, with an overall mean of 92.08% ± 2.22.

From these results, we can conclude that the approach exhibits strong and consistent performance across all folds, with minor variations in performance metrics. The standard deviation is low, which indicates stability and low variance in the results. The high recall and precision scores suggest that the model maintains a strong balance between detecting positive cases and minimizing false positives.

**Figure 4:**
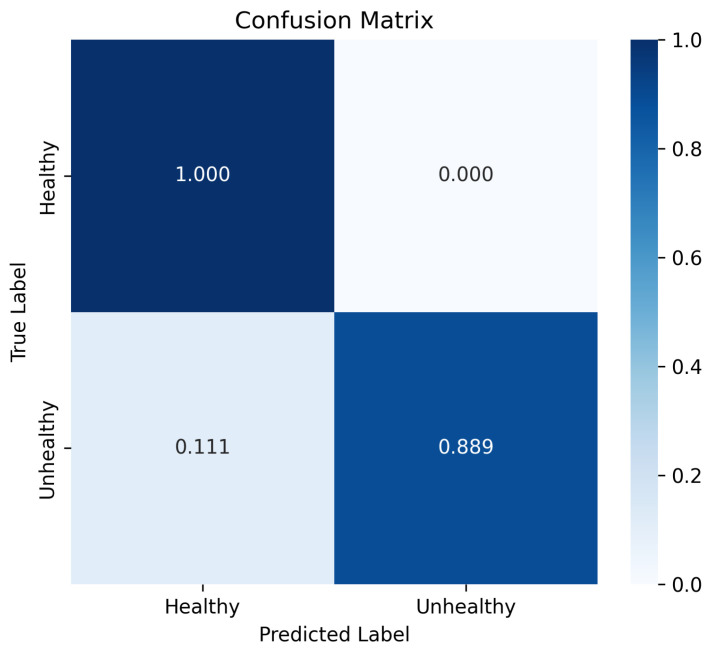
Confusion matrix of the proposed approach: a visual representation of the classification performance for healthy and unhealthy cases in Fold 1.

Table 4 presents a class-wise breakdown of evaluation metrics for Fold 1, offering a detailed view of the model’s discriminative ability to identify healthy and unhealthy cases within an imbalanced dataset. The confusion matrix, illustrated in Figure 4, further complements this analysis by depicting normalized prediction values across the two classes. This visualization helps to understand the distribution of the predictions and emphasizes the overall effectiveness of the model in distinguishing between classes. The presence of a false positive in the confusion matrix (where an unhealthy case is misclassified as healthy) is justified by the class imbalance present in the data. In future work, our aim is to refine the model by introducing additional training samples to further reduce these misclassifications.

**Figure 5:**
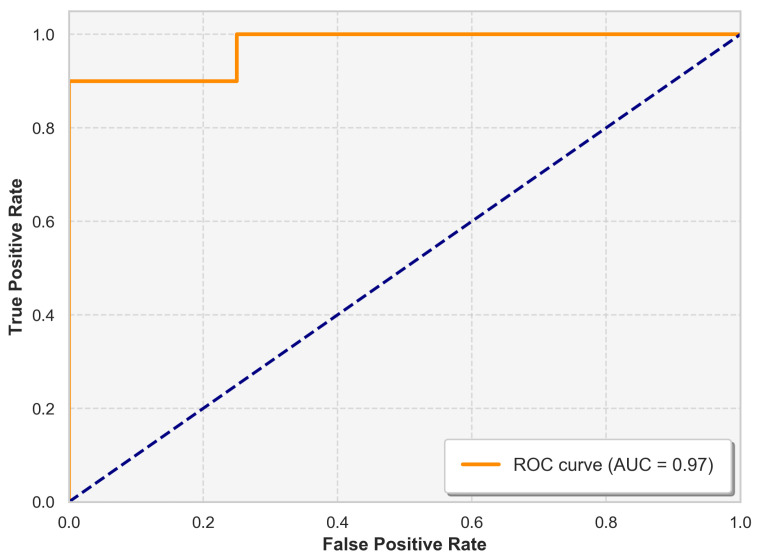
ROC curve illustrating the trade-off between the true positive rate (sensitivity) and the false positive rate for the classifier.

The ROC curve, presented in Figure 5, offers a visual representation of the performance of the model in Fold 1 by plotting the true positive rate (sensitivity) against the false positive rate in various classification thresholds. The model achieved an AUC value of 97%. This high AUC underscores the robustness of the model in maintaining a strong balance between sensitivity and specificity.

The results of the study highlight the significant impact of SSL on feature extraction in medical image classification. Using SSL, our approach was able to learn rich representations from the unlabeled data. The adoption of DIM as the basic SSL approach was extremely useful. This capability to extract meaningful and nuanced representations was vital in improving the RF classifier’s performance. Thus, the integration of SSL and DIM in our proposed model not only boosted the classification accuracy but also demonstrated the power of advanced feature extraction methods to transform AI-driven healthcare solutions.

### 6.4. Comparison with Baseline Models

Furthermore, a comparative analysis was performed to evaluate the performance of our proposed model against various traditional ML and transfer learning models. We used the same dataset, and the results are described in Table 5. Among supervised learning models, the Support Vector Machine (SVM), DT, RF, Logistic Regression (LR), and Convolutional Neural Network (CNN) were benchmarked. The Logistic Regression model achieved the highest accuracy of 82.14% among traditional ML methods, with a precision of 86.02% and an F1-score of 80.26%.

The CNN achieved the lowest performance with an accuracy of 78.57%. This underperformance is attributed to its tendency to overfit, particularly when trained on relatively small datasets. In such scenarios, CNNs often learn to memorize specific patterns within the training data rather than effectively capturing the generalizable features. This memorization leads to poorer performance when the model is tested on unseen data, as it struggles to generalize beyond the examples it was trained on.

The comparison was extended to transfer learning models, including VGG16, ResNet15, MobileNetV2, and InceptionV3. All transfer learning models demonstrated a similar precision of 86%, with varying degrees of precision and F1-scores, indicating their ability to generalize well in the dataset. For example, VGG16 showed the highest precision of 91%, suggesting a strong performance in correctly identifying positive cases, while MobileNetV2 and InceptionV3 maintained competitive F1-scores, reflecting a balanced performance in precision and recall.

Despite the robust performance of these models, our approach, which combines SSL using DIM for feature extraction and an RF classifier, outperformed the methods compared to a mean precision of 92.62%. This highlights the advantages of using SSL techniques for feature extraction, as the DIM model was able to learn more meaningful and richer representations from the dataset. Consequently, the use of SSL combined with the RF classifier demonstrates a significant improvement over both traditional supervised learning and transfer learning approaches.

## 7. Discussions

The evaluation metrics indicate that the proposed model shows immense potential to classify sinus diseases. The combination of DIM for self-supervised feature learning and the RF classifier has proven to be highly effective in capturing both global and local features. With an accuracy exceeding 90%, the model demonstrates a strong overall performance in correct classification. However, in a medical context, it is crucial to examine the precision, recall, and F1-score more closely, as false positives and negatives carry different levels of risk. The high precision (90.91%) for the unhealthy class suggests that the model effectively minimizes false positives, crucial to avoid unnecessary medical procedures. The recall of 100% for the unhealthy class indicates that the model excels in identifying patients with sinus disease, reducing the risk of missed diagnoses. The F1-score, which balances precision and recall, provides a more comprehensive view of model performance. An F1-score of 0.9524 for the unhealthy class shows that the model effectively balances identifying positive cases (high recall) with avoiding misclassification of healthy cases (high precision).

The ROC curve and the AUC value of 97% further demonstrate the strength of the model in differentiating between healthy and unhealthy cases. A high AUC indicates that the model consistently achieves a high true-positive rate while maintaining a low false-positive rate across various classification thresholds. This is particularly valuable in medical diagnostics, where both sensitivity and precision are crucial.

A notable advantage of the SSL approach was its ability to use unlabeled data, minimizing the need for extensive manual annotation by scientific personnel. This strategy not only conserved valuable time and resources but also mitigated potential privacy concerns associated with labeled data.

Despite the limited availability of labeled data, the SSL models demonstrated remarkable efficacy. These models successfully extracted meaningful patterns and representations from the unbalanced dataset, underscoring the robustness of this approach in suboptimal data environments. The successful implementation of SSL in this study accentuates its potential as a powerful tool for researchers confronted with imperfect or limited datasets, particularly in domains where data acquisition is challenging or privacy considerations are paramount.

In this context, the importance of privacy-preserving techniques cannot be overstated, especially when dealing with sensitive medical imaging data such as CT and MRI scans. Our study used anonymized public data. However, future clinical applications of this proposed framework should integrate lightweight encryption schemes and privacy-sensitive AI models to safeguard patient information. Techniques such as bit-plane decomposition with chaos-based encryption [45] and privacy-preserving DL models [46,47] offer promising directions. Incorporating such strategies into future iterations of our system will help ensure secure data handling, storage, and transmission across real-world clinical deployments.

This study demonstrates promising results in the binary classification of sinonasal conditions. However, we acknowledge several important limitations and future directions. A significant limitation of our current binary classification system is its inability to differentiate between chronic and acute cases of sinus disease. This distinction is particularly challenging, as it fundamentally requires temporal information. While the model achieved an accuracy of 92.62% on the internal dataset, it has not yet been evaluated on external datasets from different sources or imaging conditions. This limits the ability to assess its generalization in real-world clinical settings.

Furthermore, the relatively small size dataset and the lack of external validation may introduce bias and limit the robustness of the results, especially when applied to diverse patient populations or imaging protocols. In addition, the current binary classification (healthy vs. unhealthy) represents a simplified approach to the complex spectrum of sinonasal pathologies. There are multiple types of disease with significant visual polymorphism. In clinical practice, differentiation between specific conditions (e.g., inflammatory diseases, neoplasms, fungal infections) is crucial for proper patient management and requires more granular classification.

To overcome these limitations, future research should focus on the following:

1. Innovative approaches for increasing sample size used by the proposed classification model: GANs are capable of producing artificial images, particularly for uncommon diseases like sinonasal pathologies.
2. Expanding the dataset to include diverse pathological manifestations with detailed subtype annotations: This can be addressed by including a wider range of sinonasal conditions like CRS, allergic rhinitis, benign and malignant tumors, fungal infections such as allergic fungal RS, and rare diseases such as tuberculosis, sarcoidosis, and Wegener’s granulomatosis in future datasets.
3. Incorporating relevant clinical data and patient history to enhance diagnostic accuracy: For diseases such as acute versus chronic sinonasal pathology, where patient history and disease evolution are important for diagnosis, imaging alone does not provide a complete clinical context.
4. Integrating sequential imaging data to capture disease progression and distinguish between acute and chronic cases: Future models should take into account sequential imaging data, such as CT or MRI images collected at different phases, to track changes over time and identify patterns of evolution, such as bone involvement, sinus opacification, and mucosal thickening.
5. Enhancing explainability and clinical interpretability: Future work will also explore explainability techniques such as Grad-CAM, SHAP, or attention-based visualization to highlight the key regions influencing model decisions. This will support integration into clinical workflows by allowing radiologists and medical practitioners to interpret the model’s outputs in a transparent and informative manner.

## 8. Conclusions

This study presents a novel AI-based system for the detection of sinonasal pathology using SSL techniques and RF algorithms. This research makes several significant contributions to the field. Firstly, a new collection of images comprising 137 CT and MRI images carefully labeled by expert radiologists was investigated. Although limited, this dataset serves as a valuable resource for future research in AI-based sinus disease classification. Furthermore, our study employs a unique combination of the DIM model for self-supervised feature extraction and an RF classifier. This approach effectively captures global and local features from imaging data, demonstrating high efficacy in classifying healthy and pathological cases. The performance evaluation of the proposed model achieved impressive results, with a mean classification accuracy of 92.62%. This study successfully used SSL techniques to overcome the challenges posed by an unbalanced dataset. This approach minimized the need for extensive manual annotation, conserved resources, and addressed potential privacy concerns.

Future research will focus on validating the model using external datasets from different institutions to better assess its generalization performance in varying imaging conditions and scanner settings. Incorporating cross-institutional datasets and multimodal imaging data will be a key step toward improving the clinical applicability of the model and ensuring greater reliability.

## Figures and Tables

**Table 1:** Detailed overview of recent AI innovations for sinus disorders.

Refs.	AI Application	Used Model	Key Results
Ding et al. [16]	Various ML algorithms (ResNet, InceptionV3, Unet) for OM diagnosis	Literature review of AI applications in OM, focusing on machine learning algorithms for diagnosis	- AI shows promise in accurate detection of OM - ML algorithms successfully applied to OM diagnosis - Potential for automated image interpretation and outcome prediction
Huang et al. [20]	3D distance-guided network on CBCT images	Two-stage model (1) Volumetric regression: network (2) 3D distance-guided network for classification	- Mean MRE for landmark detection 0.87 mm - Accuracy: 97% - Sensitivity: 0.92 - Specificity: 98% - AUC: 95%
He et al. [21]	Multi-task DL network for sinus segmentation and CRS recurrence prediction	Developed and validated DL radionics-based nomogram using 265 paranasal sinuses CT images from two medical centers	- Achieved good sinus segmentation results - Nomogram combining DL signature and clinical factors showed excellent recurrence prediction ability
Bu et al. [22]	Dynamic Uncertain Causality Graph (DUCG) theory-based system for sore throat diagnosis	Established a sore throat DUCG model as the diagnostic knowledge base, integrating epidemiological data, knowledge, and clinical experience	- System can diagnose 27 sore throat-related diseases - 100% accuracy in the initial test with 81 cases - 98% accuracy in third-party hospital verification - 99.9% recognition by doctors in primary hospitals
Doğan and Bor [24]	AI model for distinguishing four GERD phenotypes per patient	Developed and tested GERD phenotype algorithm on 2052 patients and CC 3.0 algorithm on 133 patients	- 100% accuracy for both GERD phenotyping and CC3.0 in tests - System warns physicians of wrong phenotyping decisions - Annual number of cured patients doubled (from 400 to 800) since system implementation in 2017
Liu et al. [25]	Patient-specific, learning-based method for 3D reconstruction	Comparison of method with Structure from Motion, dense reconstruction from COLMAP, and ground truth anatomy from CT	- Produced watertight, textured reconstructions - Enabled measurement of clinically relevant parameters in good agreement with CT
Zhou et al. [26]	Various ML methods for classification and prediction of olfactory dysfunction in CRS	Comparison of four ML methods with traditional logistic regression using data from 611 CRS patients	- 34% of patients demonstrated - ML methods performed favorably compared to logistic regression - Identified several predictors, including socioeconomic factors

**Table 2:** Hyperparameters used to configure the DIM and RF models.

Model Components	Parameters	Values
DIM	Batch Size	32
	Learning Rate	0.0001
	Epochs	160
	Global Discriminator Weight	0.5
	Local Discriminator Weight	1.0
	Prior Discriminator Weight	0.1
	Optimizer	Adam
RF	Number of Trees	100
	Criterion	Gini Impurity
	Max Depth	None
	Min Samples Split	2
	Min Samples Leaf	1
	Bootstrap	True

**Table 3:** Five-fold cross-validation results.

Fold	Accuracy (%)	Precision (%)	Recall (%)	F1-Score (%)
Fold 1	91.2	90.5	91.0	90.7
Fold 2	89.5	88.9	89.2	89.0
Fold 3	95.1	94.3	95.0	94.6
Fold 4	93.6	92.7	93.1	92.9
Fold 5	93.7	93.0	93.5	93.2
Mean ± Std	92.62±2.24	91.88±2.15	92.36±2.27	92.08±2.22

**Table 4:** A class-wise breakdown of evaluation metrics for Fold 1.

Class	Precision (%)	Recall (%)	F1-Score (%)
Healthy	100	88.9	90.71
Unhealthy	90.91	100	95.24

**Table 5:** Experimental results comparing the other methods against our approach.

Model	Type of Learning	Accuracy (%)	Precision (%)	Recall (%)	F1-Score (%)
SVM	Supervised learning	78 ± 1.2	79 ± 1.0	78 ± 1.3	76 ± 1.1
DT	Supervised learning	75 ± 1.5	74 ± 1.4	75 ± 1.6	73 ± 1.2
RF	Supervised learning	78 ± 1.3	84 ± 1.7	79 ± 1.2	76 ± 1.5
LR	Supervised learning	82 ± 1.1	86 ± 1.5	82 ± 1.2	80 ± 1.4
CNN	Supervised learning	79 ± 1.4	89 ± 1.9	79 ± 1.3	80 ± 1.6
VGG16	Transfer learning	86 ± 1.0	91 ± 1.3	86 ± 1.1	87 ± 1.2
ResNet15	Transfer learning	86 ± 1.2	90 ± 1.4	86 ± 1.2	86 ± 1.3
MobileNetV2	Transfer learning	86 ± 1.1	89 ± 1.2	86 ± 1.0	85 ± 1.2
InceptionV3	Transfer learning	86 ± 1.2	88 ± 1.3	86 ± 1.1	86 ± 1.3
Proposed Approach	SSL	92.62±2.24	91.88±2.15	92.36±2.27	92.08±2.22

## Data Availability

The dataset used in this study is publicly available at the GitHub repository [41].

## Acronym List

A list of acronyms defined throughout the paper

**Acronym**	**Full Words**
AI	Artificial Intelligence
SSL	Self-Supervised Learning
RF	Random Forest
DIM	Deep InfoMax
CRS	Chronic Rhinosinusitis
ML	Machine Learning
DL	Deep Learning
OM	Otitis Media
RS	Rhinosinusitis
CBCT	Cone Beam Computed Tomography
GERD	Gastroesophageal Reflux Disease
DUCG	Dynamic Uncertain Causality Graph
XAI	Explainable AI
VAEs	Variational AutoEncoders
GANs	Generative Adversarial Networks
AUC	Area Under the Curve
SVM	Support Vector Machine
DT	Decision Tree
LR	Logistic Regression
CNN	Convolutional Neural Network
ROC	Receiver Operating Characteristic
LSTM	Long Short-Term Memory
RNNs	Recurrent Neural Networks
NLP	Natural Language Processing
